# Sleep health: an unappreciated key player in colorectal cancer

**DOI:** 10.7150/jca.107117

**Published:** 2025-03-03

**Authors:** Jiahua Liu, Qihang Yuan, Yao Zhang, Xinyu Wang, Lijun Zhai, Ruiqi Wang, Chunyu Zheng, Zhijun Hong

**Affiliations:** First Affiliated Hospital of Dalian Medical University, Dalian, China.

**Keywords:** colorectal cancer, sleep, metabolite, gut microbiota, inflammation

## Abstract

Colorectal cancer (CRC) poses a significant threat to human life and health. Global cancer prevalence data in 2022 indicated that the number of new cases of CRC was about 1.92 million and the deaths were around 900,000. A variety of risk factors, including genes and environment, can induce the occurrence of CRC. Previous studies have focused on the impact of dietary patterns on the development of CRC and have ignored sleep factors. Sleep deprivation is a common problem as people's work pressure increases. Sleep disorders can lead to metabolic and immune system dysregulation in people, contributing to the development and progression of many tumors. At present, there are few reports on the relationship between sleep disorders and tumors. Therefore, the purpose of this paper is to summarize and interpret the relationship between various sleep disorders and the onset and progression of CRC. This review is the first to investigate the possible mechanisms of sleep leading to CRC from the perspectives of metabolic reprogramming, intestinal microbiota disorders, and the release of inflammatory factors. In conclusion, this study highlights the rational sleep pattern and duration, which can help inhibit the occurrence of CRC.

## 1. Introduction

Colorectal cancer (CRC) is a significant public health concern and ranks among the most prevalent malignancies worldwide. According to global cancer statistics, CRC accounted for approximately 1.92 million new cases and 900,000 deaths in 2022^1^, making it the third most common cancer in both men and women^1^. With the continued development of emerging economies, the global incidence of CRC is projected to rise to 2.5 million new cases by 2035^2^. Gender disparities in CRC incidence and outcomes have been observed. Women over 65 exhibit higher mortality rates and lower 5-year survival rates compared to their male counterparts. Moreover, women are more likely to develop right-sided (proximal) colon cancer^3^. Addressing the rising incidence and mortality of CRC remains a pressing challenge. The incidence of CRC is influenced by a complex interplay of genetic, environmental, and lifestyle factors^4^. Established risk factors include the consumption of processed meats, alcohol, smoking, and obesity. Conversely, protective factors such as dietary fiber, whole grains, dairy products, fruits, non-starchy vegetables, and supplementation with multivitamins or vitamin D lower the risk of CRC^5^. The intricate molecular characteristics of CRC reveal its association with genetic predisposition, oncogene mutations, gut microbiota imbalances, metabolic dysfunctions, and immune system responses^6^,^7^.

While previous research has emphasized the role of lifestyle factors (such as diet and exercise) in CRC development, these factors alone do not fully account for the rising global incidence. Therefore, it is necessary to investigate other potential risk factors further. In recent years, the growing prevalence of sleep disorders, driven by increasing workloads and mental stress, has emerged as a topic of significant interest. Sleep, a fundamental physiological process constituting roughly one-third of human life, is indispensable for physical and mental well-being^8^. Adults are recommended to sleep at least 7-8 hours per night to maintain optimal health^9^. As a modifiable lifestyle factor, sleep quality is critical for overall health^10^. Poor sleep has been linked to a range of health conditions, including cardiovascular diseases, metabolic disorders, and cognitive dysfunction^10^. Emerging evidence also suggests that inadequate sleep may elevate the risk of several cancers, including CRC^11^.

However, most studies investigating the link between sleep health and cancer have predominantly focused on breast^12^-^14^, lung^15^-^17^, and gastric cancers^18^-^20^. Research examining the relationship between sleep and CRC remains limited, and the underlying mechanisms connecting poor sleep quality to CRC risk are not yet well understood^19^,^21^,^22^. Therefore, this study aims to explore the relationship between sleep and CRC, uncover the underlying mechanisms and propose strategies for CRC prevention and management through the lens of sleep health.

### 2.1 Sleep disturbances alter metabolite levels and promote CRC development

Disruptions in sleep patterns and circadian rhythms can lead to significant metabolic dysregulation, contributing to obesity, insulin resistance, and diabetes. These metabolic disturbances can affect the concentrations of various blood metabolites^23^. Melatonin, an indole hormone derived from serotonin via the tryptophan-serotonin pathway in the pineal gland, plays a critical role in regulating circadian rhythms and promoting sleep quality. Its production increases with reduced exposure to light, and disruptions in circadian rhythms can significantly decrease melatonin levels^24^,^25^. Experimental studies have shown that acute sleep deprivation disrupts the rhythmicity of plasma metabolites, leading to elevated serotonin and tryptophan levels. These changes may be attributed to the reduced secretion of melatonin under sleep-deprived conditions, resulting in an accumulation of its precursor substances^23^. Beyond its role in regulating the sleep/wake cycle and circadian rhythms, melatonin is involved in immune regulation, antioxidant defense, and tumor regulation, influencing tumor initiation, progression, and prevention^26^. Changes in melatonin levels significantly increase the vulnerability of colorectal cells to carcinogenesis. Melatonin slows CRC progression by inhibiting cell proliferation and inducing apoptosis in CRC cells^27^. A cohort study by Zhang *et al.* reported a significant inverse relationship between melatonin use and CRC risk among individuals aged 50 and above (hazard ratio [HR]=0.82, 95% confidence interval [CI]: 0.72-0.92)^28^. Furthermore, another study highlighted that melatonin is safe and well-tolerated across a broad dosage range, positioning it as a promising therapeutic candidate for CRC treatment^29^. In summary, disrupted sleep patterns elevate CRC risk by adversely affecting melatonin levels.

### 2.2 Sleep disturbances drive CRC progression by inducing gut microbiota dysbiosis

The gut microbiota (GM) comprises a complex ecosystem of microorganisms within the human digestive tract, playing a pivotal role in nutrient synthesis, immune function, and maintaining intestinal barrier integrity^30^. In adults, over 90% of gut bacteria belong to the *phyla Firmicutes*, *Bacteroidetes*, *Proteobacteria*, or *Actinobacteria*, underscoring GM's critical association with intestinal health^31^. Recent studies indicate that sleep disturbances can significantly alter the composition and balance of the GM^32^.

Obstructive sleep apnea (OSA), the most prevalent form of sleep-disordered breathing, is characterized by sleep fragmentation and intermittent hypoxia (IH)^33^. Zhang *et al.* reported significant alterations in GM diversity and abundance in humans and rodents with OSA, particularly affecting the *phyla Firmicutes* and *Bacteroidetes*^34^. Similarly, Collado *et al.* found that children experiencing chronic IH due to snoring exhibited an increased abundance of *Firmicutes* and a decreased abundance of *Bacteroidetes* in their gut^35^. Gao *et al.* corroborated these findings in mouse experiments, observing similar microbiota changes^36^. Further supporting the link between GM alterations and CRC, Flemer *et al.* conducted prospective research revealing an increased abundance of *Bacteroidetes* Cluster 2 and *Firmicutes* Cluster 2 in CRC mucosa, highlighting their potential role in CRC progression^37^. Gao *et al.* further demonstrated that IH and GM dysbiosis activated hypoxia-inducible factor-1α (HIF-1α) expression and the Signal Transducer and Activator of Transcription 3 (STAT3) pathway in colonic epithelial cells, promoting OSA-related CRC development^38^. As modern society advances, shift work has become increasingly common, bringing unique sleep challenges, including difficulty initiating sleep, reduced sleep duration, and excessive daytime sleepiness^39^. A review by Reynolds *et al.* linked sleep disturbances among shift workers to GM dysbiosis, suggesting that sleep deprivation and circadian rhythm disruptions act as physiological stressors that disrupt GM composition. This dysbiosis can contribute to metabolic disorders such as obesity and type 2 diabetes^40^. Disruption of intestinal epithelial permeability due to these stressors leads to an imbalance between beneficial and pathogenic bacteria^41^, which plays a significant role in the progression of metabolic conditions such as obesity and diabetes, both high-risk factors for CRC. Moreover, increased gut epithelial permeability and bacterial translocation further contribute to CRC initiation and advancement^42^,^43^.

In an experiment conducted by Bishehsari *et al.* using mice with adenomatous polyposis coli, researchers investigated the combined effects of alcohol consumption and circadian rhythm disruption. Both groups of mice were fed an alcohol-containing diet; however, one group maintained a standard 12-hour light/12-hour dark cycle, while the other experienced weekly 12-hour phase shifts in their light/dark cycle, disrupting their circadian rhythm. The study revealed that circadian rhythm disruption substantially exacerbated alcohol-induced polyposis and CRC development. Fecal microbiota analysis revealed that circadian rhythm disturbances promoted the transition of mast cells from MCP2^+^ to MCP6^+^, thereby mediating inflammation and fostering a pro-tumor inflammatory environment^44^.

Research has demonstrated that various sleep disturbances, such as sleep disorders, insomnia, and circadian rhythm disruptions, induce significant alterations in GM composition and function^32^. Abnormal sleep patterns and durations affect the composition, diversity, and functionality of GM via the brain-gut-microbiota axis^45^. Moreover, the relationship between sleep quality and GM is bidirectional, as maintaining proper GM diversity promotes healthier sleep patterns^46^. The GM can contribute to the development of CRC by releasing various metabolites, proteins, and macromolecules that interact with the host's colonic epithelial and immune cells^47^. For instance, Okumura *et al.* demonstrated that *Porphyromonas* species promote CRC progression by secreting the bacterial metabolite butyrate, which subsequently induces cellular senescence^48^.

Emerging research continues to elucidate the mechanisms through which GM influences CRC. Specific bacteria, including *Fusobacterium nucleatum*, *Escherichia coli*, *Enterococcus faecalis*, *Streptococcus gallolyticus*, and *enterotoxigenic Bacteroides fragilis*, have been identified as closely associated with CRC initiation and progression^49^. However, it remains unclear whether poor sleep habits directly alter the composition of these specific bacteria. Nonetheless, sleep disturbances can influence GM composition at the phylum level, causing dysbiosis that elevates CRC risk.

### 2.3 Sleep disturbances induce pro-inflammatory cytokine release, fostering CRC development

The immune system is essential for maintaining health by identifying and eliminating cancerous cells through immune surveillance. Besides this primary function, other immune components, including various immune cells and regulatory factors, play a pivotal role in suppressing cancer^50^,^51^. Burgos-Molina *et al.*'s review highlights that chronic inflammation, mediated by various cells and factors such as macrophages, lymphocytes, and pro-inflammatory cytokines such as tumor necrotic factor-α (TNF-α), interleukin-6 (IL-6), and IL-1β, promotes tumor progression and alters the tumor microenvironment in multiple ways, making it a significant factor in CRC development^51^. Increasing evidence suggests that sleep deprivation impairs anti-tumor immune responses, thereby promoting cancer progression^52^.

In their review, Akkaoui *et al.* summarize the changes in several regulatory factors during sleep, noting that sleep deprivation considerably elevates the levels of pro-inflammatory cytokines (such as IL-1, IL-6, and TNF-α)^53^. A study tracking 2,500 older individuals over 7 years found that sleeping less than 5 hours a night was associated with elevated levels of pro-inflammatory cytokines (such as IL-6 and TNF-α) and CRP^54^. Furthermore, both insufficient and excessive sleep can lead to increased levels of pro-inflammatory cytokine levels. Patel *et al.* investigated the sleep patterns of 614 individuals and found that the average habitual sleep duration was 7.6 hours. As sleep duration increased, so did the levels of CRP and IL-6. Each additional hour of habitual sleep was associated with an 8% rise in CRP levels and a 7% increase in IL-6 levels. Conversely, for every hour of sleep reduction, TNF-α levels increased by an average of 8%^55^. Interleukins (ILs) can promote CRC development through various mechanisms, including tumor initiation, growth, angiogenesis, and metastasis^56^. Interleukin-6 (IL-6) plays a crucial role in nearly every stage of CRC progression. Upon binding to glycoprotein 130 (gp130), IL-6 primarily regulates CRC development through three signaling pathways: Src Homology 2 Domain-containing Phosphatase 2 - Rat Sarcoma - Extracellular Signal-Regulated Kinase (Shp2-Ras-ERK), Janus Kinase 1/2 (JAK1/2)-STAT3, and Phosphoinositide 3-Kinase - Protein Kinase B - Mechanistic Target of Rapamycin (PI3K-Akt-mTOR)^56^. Furthermore, a meta-analysis by Liu *et al.* identified IL-1 family members, including IL-1α, IL-1β, and Interleukin-1 Receptor Antagonist Variable Number Tandem Repeats (IL-1RN VNTR), as significant genes associated with CRC susceptibility^57^. The TNF facilitates CRC progression by activating the nuclear factor-kappa B (NF-κB) pathway, which supports cancer cell survival and induces gene expression in invasion and metastasis^58^.

Bao *et al.*'s mouse model experiment demonstrated that sleep deprivation increases gamma-aminobutyric acid (GABA) levels in peripheral blood, promoting CRC cell proliferation and migration through the endogenous regulation of miR-223-3p. Furthermore, microRNA miR-223-3p enters macrophages via exosomes, activating the mitogen-activated protein kinase (MAPK) pathway, inducing M2 polarization, and stimulating the secretion of IL-17, further accelerating tumor cell proliferation and migration^59^.

**Figure 1:**
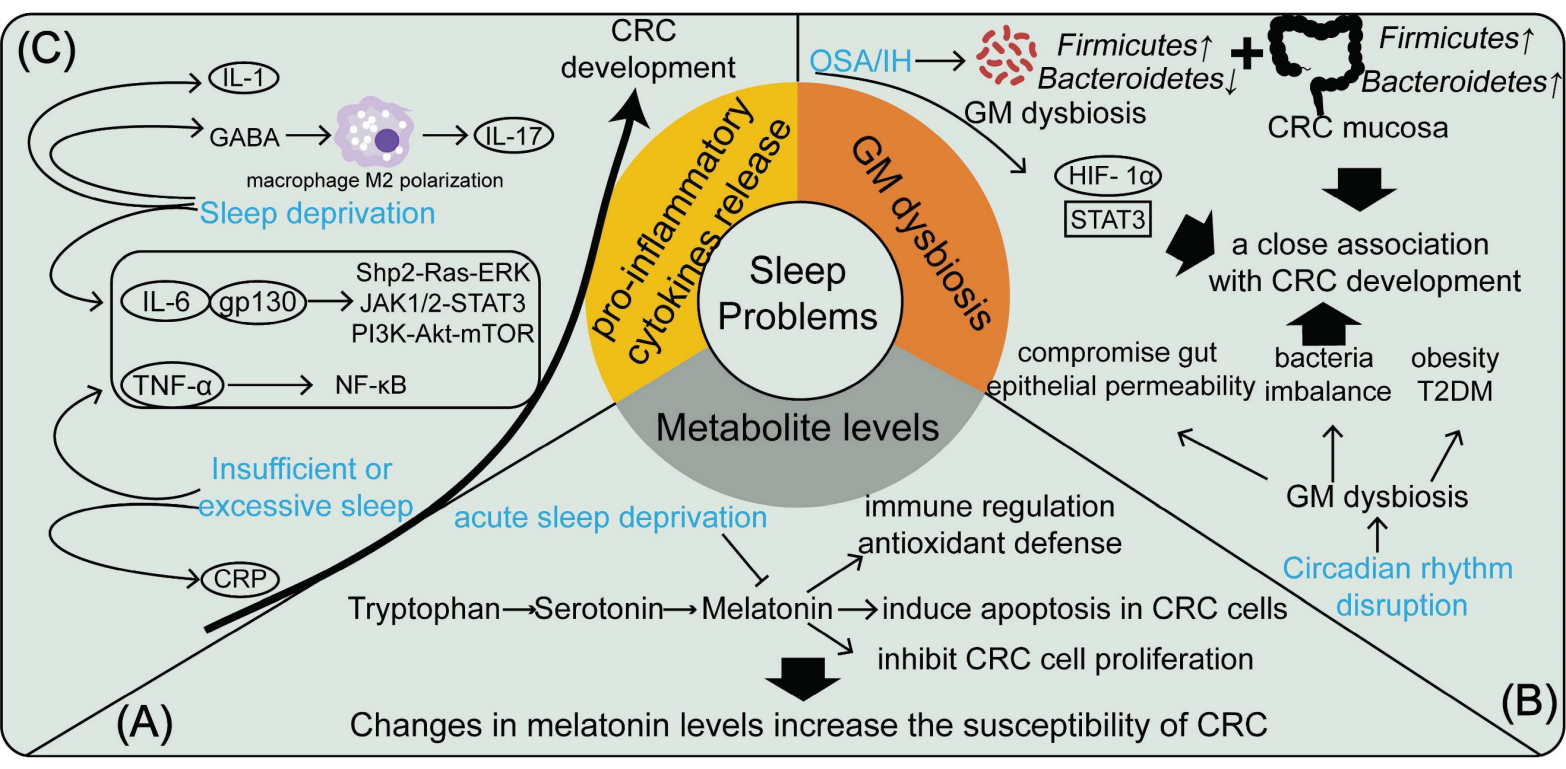
The mechanisms by which sleep problems contribute to the development of CRC are manifested in changes in metabolite levels, GM flora dysbiosis, and the release of pro-inflammatory cytokines. (A) Acute sleep deprivation causes changes in melatonin levels, which increases CRC susceptibility; (B) OSA/IH and circadian rhythm disruption, as physiological stressors, disrupt the gut microbiota and are closely associated with CRC development; (C) Sleep deprivation and insufficient or excessive sleep cause the release of multiple pro-inflammatory factors in the body, which promotes CRC development.

In summary, sleep problems elevate CRC risk by influencing the release of pro-inflammatory cytokines and promoting systemic inflammation (Figure 1).

## 3. Research on the correlation between sleep patterns and CRC

Sleep can be assessed across dimensions, including sleep quality, duration, efficiency, timing, and alertness^60^. Sleep quality and duration are particularly significant in evaluating sleep status^61^. Changes in sleep quality can trigger a series of physiological responses, with sleep disorders being a major contributing factor. The following sections explore the relationship between poor sleep habits and CRC from four perspectives.

### 3.1 Correlation between sleep disorders and CRC

Sleep disorders are crucial indicators of sleep quality. According to the third edition of the International Classification of Sleep Disorders, sleep disorders are categorized into insomnia, sleep-related breathing disorders (such as OSA), hypersomnia, circadian rhythm sleep-wake disorders, and parasomnias^62^. Sleep disorders have been linked to various physical and mental health issues^10^. Emerging evidence suggests a potential association between sleep disorders and cancer development^63^. Several studies have specifically identified a higher risk of CRC in individuals with sleep disorders^11^,^18^,^64^,^65^. A population-based nested case-control study reported that individuals with sleep disorders faced a significantly elevated risk of CRC compared to those without such disorders (odds ratio [OR]=1.29, 95% CI: 1.13-1.47). Moreover, patients with sleep disorders and depression exhibited an alarming 5.69-fold increased risk of CRC compared to the control group^65^. Liu *et al.*^66^ employed tools such as the Pittsburgh Sleep Quality Index (PSQI), Perceived Stress Scale (PSS10), Multidimensional Scale of Perceived Social Support (MSPSS), and Connor-Davidson Resilience Scale (CD-RISC) to examine the interplay between sleep quality, psychological factors (such as stress, resilience, and social support), and the risk of precancerous conditions of CRC (PCRC). Their findings revealed that a PSQI score indicative of moderate to severe sleep disorders (≥2) was significantly associated with an increased risk of PCRC (OR=20.15, 95% CI: 4.22-96.26).

### 3.2 Correlation between insomnia and CRC

Insomnia, a sleep continuity disorder, is characterized by difficulties initiating or maintaining sleep, often resulting in daytime symptoms such as excessive fatigue, physical discomfort (headaches or body pain), mood disturbances, cognitive or occupational impairment, and dissatisfaction with sleep quality. Diagnosis requires evidence of sleep-related challenges that lead to functional impairments during the day despite adequate opportunities for rest^67^,^68^. Insomnia has been linked to an increased risk of several health conditions, including depression, dementia, non-alcoholic fatty liver disease, hypertension, and cardiovascular diseases^69^. Furthermore, epidemiological studies have consistently suggested a correlation between insomnia and cancer^70^-^72^. A recent meta-analysis reported that individuals with insomnia have a 24% increased overall risk of developing cancer^71^. Mechanisms potentially underlying this association include disruptions in melatonin, circadian rhythm disruptions, and imbalances in appetite-regulating hormones (such as leptin and ghrelin)^71^. The relationship between insomnia and CRC risk remains controversial. A study conducted in Taiwan identified a substantially higher risk of colon cancer in patients with insomnia^70^, and findings from a nested case-control study supported an increased risk of CRC in individuals with insomnia^65^. However, contrasting evidence from a large nationwide cohort study in Korea indicated a decreased risk of CRC among patients with insomnia, regardless of gender^72^.

### 3.3 Correlation between obstructive sleep apnea (OSA) and CRC

OSA is a sleep disorder characterized by recurrent episodes of complete or partial upper airway obstruction during sleep, leading to apnea or hypopnea^73^. This condition is associated with intermittent hypoxia, sleep fragmentation (SF), and heightened sympathetic nervous activity. Common clinical manifestations include loud snoring, excessive daytime sleepiness, and cognitive impairment^73^. Research has highlighted OSA's significant role in cancer development, with its effects primarily attributed to intermittent hypoxemia, SF, or both^74^. These factors can disrupt standard cellular transcription by altering sympathetic nerve activity, promoting angiogenesis, activating inflammatory pathways, and modulating immune cell behavior. Such changes may enhance tumor aggressiveness, invasiveness, and resistance to treatment^75^,^76^. Hypoxic environments caused by OSA may contribute to CRC progression^77^,^78^. A population-based retrospective cohort study revealed that individuals with OSA had a significantly higher risk of developing CRC than non-OSA individuals (adjusted HR=1.80, 95% CI: 1.28-2.52). Moreover, the risk appeared to increase with the frequency of medical visits related to OSA^77^. However, a meta-analysis by Niranjan *et al.* noted that while biological mechanisms suggest a potential link, there is insufficient evidence to establish OSA as a direct risk factor for CRC conclusively. Further studies are required to clarify this relationship^79^.

### 3.4 Correlation between Sleep Duration and CRC

Sleep duration has emerged as a potential risk factor influencing the incidence and mortality of various chronic diseases^80^-^82^. Both long sleep duration (typically defined as ≥9 hours) and short sleep duration (typically defined as ≤6 hours) have been associated with increased risks of cardiovascular disease^83^,^84^, type 2 diabetes^85^,^86^, obesity^86^,^87^, and cancer^22^,^88^. However, the association between sleep duration and CRC is inconsistent. Some studies suggest a U-shaped relationship on the graph, indicating that short and long sleep durations may elevate CRC risk^89^. Other studies report an association with only long sleep duration^22^,^90^ or short sleep duration^91^-^93^, while some research indicates no considerable association between sleep duration and CRC risk^94^.

Prolonged sleep duration has been associated with a 21% increase in CRC risk^16^, potentially due to heightened levels of systemic inflammation^95^, a critical factor in cancer development and progression^96^. A meta-analysis by Zhao *et al.*^88^ examining cohort studies further supported this finding, identifying a positive correlation between sleep duration and CRC risk (HR=1.29, 95% CI: 1.09-1.52). A case-control study on self-reported sleep duration revealed that long sleep duration (≥9 hours) was associated with an increased risk of CRC compared to the reference duration of 7 hours^19^. Zhang *et al.*^90^ conducted a 22-year follow-up of 30,121 men and 76,368 women for 22 years. They found that compared to an average sleep duration of 7 hours, a significant association was observed between long sleep duration and CRC risk in men (HR=1.35, 95% CI: 1.00-1.82), with a weaker association in women (HR=1.11, 95% CI: 0.85-1.44). This risk was particularly notable in individuals who were overweight or frequently snored. Similarly, a meta-analysis by Lu *et al.*^97^ of 10 studies (involving 8,392 cases and 555,678 participants) confirmed a positive correlation between sleep duration and CRC risk (relative risk [RR]=1.29, 95% CI: 1.09-1.52).

Conversely, short sleep duration has been linked to a 54% increase in CRC-related mortality^93^. Disrupted circadian rhythms^98^, reduced nocturnal melatonin production^63^, and impaired immune function^99^ associated with insufficient sleep may contribute to cancer progression. Furthermore, short sleep is linked to overweight and obesity^100^, which elevate cancer risk through mechanisms such as inflammation and insulin resistance^101^,^102^. Thompson *et al.*^92^ reported a significant association between short sleep duration and the occurrence of colorectal adenomas. Individuals sleeping fewer than 6 hours per night exhibited a nearly 50% increased risk of developing colorectal adenomas compared to those sleeping at least 7 hours (OR=1.47, 95% CI: 1.05-2.06), highlighting short sleep duration as a potential risk factor for early-onset CRC. In a national randomized clinical trial evaluating patients with resected stage III colon cancer receiving standardized treatment and follow-up, hazard ratios for sleep durations of ≤5 hours and ≥9 hours were 2.14 (95% CI: 1.14-4.03) and 2.34 (95% CI: 1.26-4.33), respectively. These findings underscore the significant association between extreme sleep durations and increased mortality risk^103^. Therefore, maintaining an optimal sleep duration is crucial for preventing CRC and improving patient outcomes.

### 3.5 Correlation between napping and CRC

Beyond nighttime sleep, napping is an important indicator of overall sleep health^104^. However, research on the relationship between napping and cancer remains limited and inconsistent. Some studies suggest that the absence of napping may elevate cancer risk. For instance, a 2023 study reported that participants who did not nap faced a 60% higher cancer risk compared to those napping over 60 minutes (HR=1.69, 95% CI: 1.01-2.55), with the risk further increasing in those who combined non-napping with short nighttime sleep^105^. Another study focusing on men revealed that not napping during the day was associated with a higher cancer risk compared to napping for 1-30 minutes (HR=2.03, 95%CI: 1.01-4.13)^106^.

Conversely, other studies suggest that frequent or prolonged naps may increase cancer risk. For instance, a 2021 case-control study reported that napping frequently (6-7 days/week) and for extended durations (over 60 minutes) naps were associated with an increased CRC incidence^19^. Similarly, an analysis of 4,869 patients with CRC revealed that daily naps lasting 1 hour or more were associated with higher overall and cardiovascular disease-related mortality. However, no significant association was observed with CRC-specific mortality^93^.

Overall, the impact of napping on cancer risk, particularly its relationship with CRC, remains unclear and warrants further investigation.

### 3.6 Correlation between sleep-wake patterns and CRC

Sleep timing (encompassing bedtime and wake-up time) reflects an individual's sleep-wake preference and schedule. Recent studies have highlighted the association between sleep timing and adverse health outcomes, linking later bedtimes to negative effects. For instance, research indicates that individuals who fall asleep between 10:00 and 11:00 PM have the lowest risk of cardiovascular disease, while those going to bed at midnight or later experience the highest incidence^107^. Similarly, a community-based study of Hispanic/Latino adults aged 18-74 found that later bedtimes were correlated with increased insulin resistance^108^.

Although limited research exists on the direct relationship between sleep timing and cancer risk, studies have investigated the role of sleep chronotype in cancer. Sleep chronotype, an essential characteristic of sleep patterns, reflects an individual's preferred timing for sleep and wakefulness. Chronotypes are generally classified into three categories: morning type (prefers early sleep and rise), evening type (late sleep and late rise), and intermediate type, with morning and evening types representing the extremes of this spectrum.

**Figure 2:**
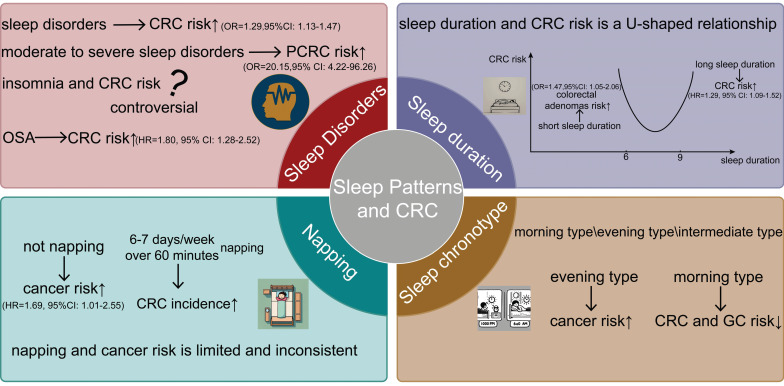
Through the four dimensions of sleep disorders, sleep time, naps, and sleep patterns, their relationship with the occurrence and development of CRC is presented from an epidemiological perspective. Sleep disorders (insomnia, OSA), too long or too short sleep duration, the presence or absence of napping, and the type of sleep chronotypes all affect the incidence of CRC.

Evening chronotypes are associated with lower physical activity^109^ and poor dietary choices^110^, which may contribute to adverse health effects and a heightened cancer risk. Conversely, morning types appear to lower the risk of certain cancers. Research has shown a significant association between morning types and a reduced risk of breast^13^ and prostate cancers^111^. Yuan *et al.* conducted a Mendelian randomization study using data from the UK Biobank and Finnish databases to assess the association between gastrointestinal cancers and sleep chronotype. They identified an inverse correlation between morning types and the risk of gastrointestinal cancers, particularly CRC and gastric cancers^20^ (Figure 2).

## 4. Conclusion

The incidence and mortality rates of CRC remain alarmingly high, posing a significant health threat. While the prognosis of CRC has slightly improved with the widespread use of colonoscopy and the removal of precursor lesions, the incidence of CRC is rising among younger individuals in some high-income countries. It is well-established that unhealthy lifestyle factors, such as smoking, excessive alcohol consumption, sedentary behavior, and high-fat, low-fiber diets, increase the risk of CRC. However, with increasing mental stress, sleep disturbances have become more prevalent. Numerous studies have established a correlation between sleep patterns and cancer, suggesting that poor sleep may elevate cancer risk and influence the development and prognosis of CRC.

This review examines how inadequate sleep contributes to CRC progression through metabolic changes, GM dysbiosis, and the release of pro-inflammatory cytokines. Disrupted sleep habits impact melatonin production, alter the gut microbiota by disrupting the balance of *Firmicutes* and *Bacteroidetes*, and promote inflammation by triggering cytokines (such as IL-1, IL-6, and TNF-α), all of which support CRC development. Furthermore, this review discusses the association between sleep patterns and CRC from an epidemiological perspective, focusing on sleep disorders, sleep duration, napping, and sleep-wake cycles. Improving sleep quality, maintaining a balanced sleep duration, adopting a regular napping routine, and establishing early sleep and wake habits can significantly lower CRC risk.

Research on the correlation between sleep habits and CRC is limited, and the topic remains controversial. Moreover, the lack of randomized controlled trials (RCT) establishes a direct causal relationship between sleep disorders and CRC in the general population. Most existing studies are descriptive and observational, failing to explore the underlying mechanisms connecting poor sleep habits to CRC. These gaps present ongoing challenges in this field. However, future research is expected to provide deeper insights into the relationship between sleep habits and CRC, offering new avenues for early prevention and effective management of the disease.
